# The last giants: New evidence for giant Late Triassic (Rhaetian) ichthyosaurs from the UK

**DOI:** 10.1371/journal.pone.0300289

**Published:** 2024-04-17

**Authors:** Dean R. Lomax, Paul de la Salle, Marcello Perillo, Justin Reynolds, Ruby Reynolds, James F. Waldron

**Affiliations:** 1 Palaeobiology Research Group, School of Earth Sciences, University of Bristol, Bristol, United Kingdom; 2 Department of Earth and Environmental Sciences, The University of Manchester, Manchester, United Kingdom; 3 The Etches Collection – Museum of Jurassic Marine Life, Dorset, United Kingdom; 4 Paleontology division, Institute of Geosciences, University of Bonn, Bonn, Germany; 5 Braunton, Devon, United Kingdom; 6 Dinosaurs Will Always Be Awesome, DWABA Museum, Orlando, Florida, United States of America; University of Silesia, POLAND

## Abstract

Giant ichthyosaurs with body length estimates exceeding 20 m were present in the latest Triassic of the UK. Here we report on the discovery of a second surangular from the lower jaw of a giant ichthyosaur from Somerset, UK. The new find is comparable in size and morphology to a specimen from Lilstock, Somerset, described in 2018, but it is more complete and better preserved. Both finds are from the uppermost Triassic Westbury Mudstone Formation (Rhaetian), but the new specimen comes from Blue Anchor, approximately 10 km west along the coast from Lilstock. The more complete surangular would have been >2 m long, from an individual with a body length estimated at ~25 m. The identification of two specimens with the same unique morphology and from the same geologic age and geographic location warrants the erection of a new genus and species, *Ichthyotitan severnensis* gen. et sp. nov. Thin sections of the new specimen revealed the same histological features already observed in similar giant ichthyosaurian specimens. Our data also supports the previous suggestion of an atypical osteogenesis in the lower jaws of giant ichthyosaurs. The geological age and giant size of the specimens suggest shastasaurid affinities, but the material is too incomplete for a definitive referral. *Ichthyotitan severnensis* gen. et sp. nov., is the first-named giant ichthyosaur from the Rhaetian and probably represents the largest marine reptile formally described.

## Introduction

Ichthyosaurs were the first marine tetrapods to attain giant size. The recent discovery of *Cymbospondylus youngorum* from the early Middle Triassic (Anisian, ~245 Ma) of Nevada, USA, estimated to be around 15–17 m in length, shows that ichthyosaurs reached giant body size as little as 3–5 million years after their first appearance [1, 2]. It was during the Late Triassic, however, when the largest known ichthyosaurs emerged, belonging to the family Shastasauridae. These include the Carnian (~230 Ma) *Shonisaurus popularis* (16 m) [3, 4] and middle Norian (~215 Ma) *Shonisaurus sikanniensis* (21 m) [5]. The remains of giant ichthyosaurs from the Swiss Late Triassic have also been described recently, including what is believed to be the largest ichthyosaur tooth yet discovered [6].

In 2018, Lomax et al. [7] reported on a large, isolated jaw fragment (an incomplete left surangular) from a giant ichthyosaur collected by PdlS in 2016 from the latest Triassic (Rhaetian) in the UK, which provided a reassessment of the purported Aust ‘dinosaurian’ bones that were reinterpreted to belong to the jaws of giant Rhaetian ichthyosaurs. As discussed in Lomax et al., body size estimates (ranging from 22–30+ metres) based on these isolated bones suggest that they probably represent the largest ichthyosaurs known to date, larger even than *S*. *sikanniensis*.

This work reports the discovery of another large surangular from a giant Triassic (Rhaetian) ichthyosaur in the UK. The new specimen is a right surangular that is morphologically identical but more complete than the left surangular described by Lomax et al. [7]. A combination of unique morphological characters observed in both examples suggest that they represent a new taxon. Both specimens were discovered in Somerset, UK, and were collected from strata dating to the latest Rhaetian, approximately 202 Ma [7, 8]. The stratigraphic horizon present immediately above the level of both finds represents a period of cataclysm with extensive seismite and tsunamite rocks [9, 10], indicative of the Late Triassic global mass extinction event. This extinction greatly reduced both ecomorphological disparity and species diversity and is considered the most poorly understood of the ‘Big Five’ mass extinctions [11–14]. It is probable that this lineage of giant ichthyosaurs vanished during the end-Triassic mass extinction event, and that ichthyosaurs never reached this size again before their extinction in the early Late Cretaceous (Cenomanian), around 94 Ma [15].

## Materials and methods

**Fig 1:**
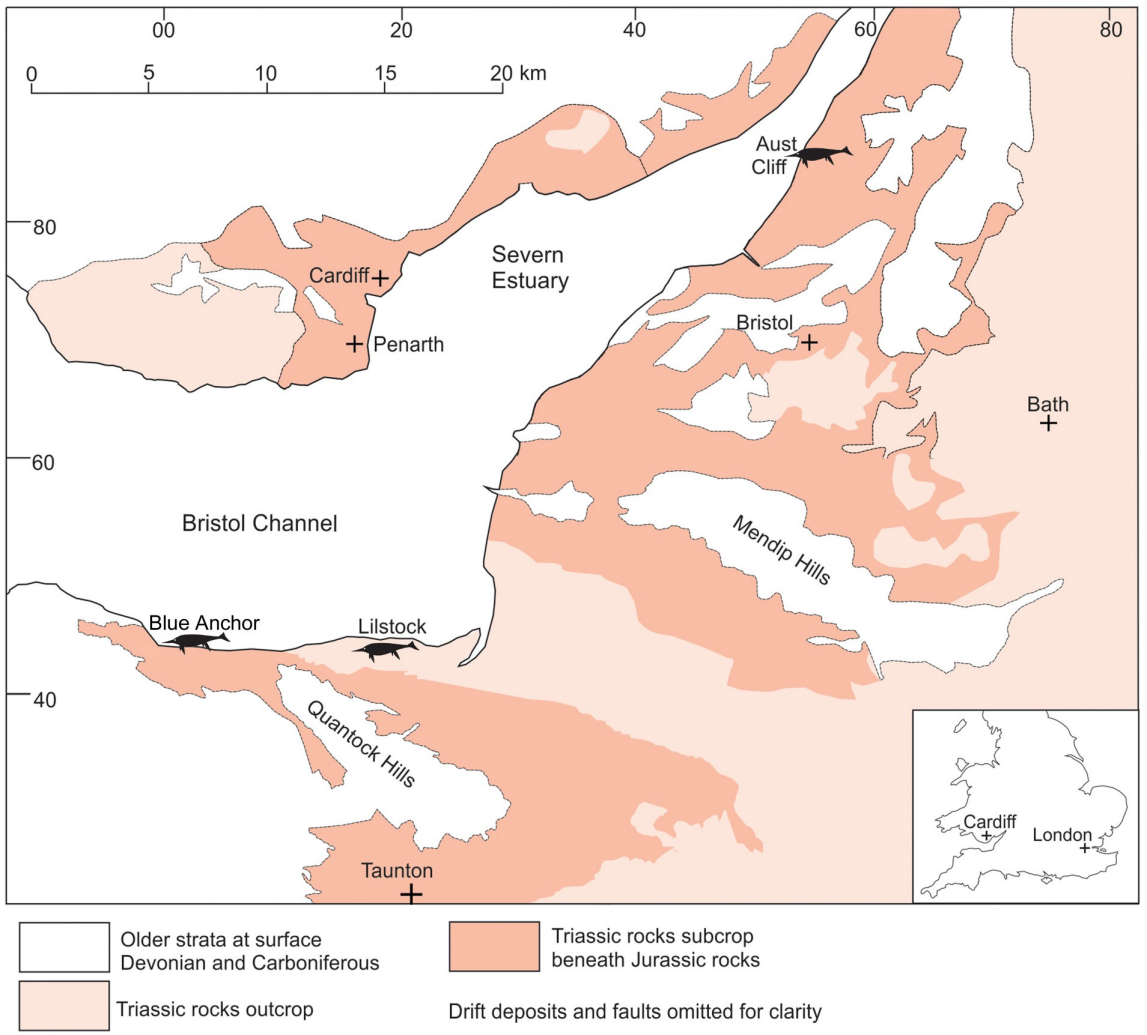
Distribution of the Triassic rocks in the Bristol Channel–Severn Estuary area and the three key ichthyosaur localities (where specimens discussed herein were found) referred to in the text. Modified from Lomax et al. 2018 [7].

**Fig 2:**
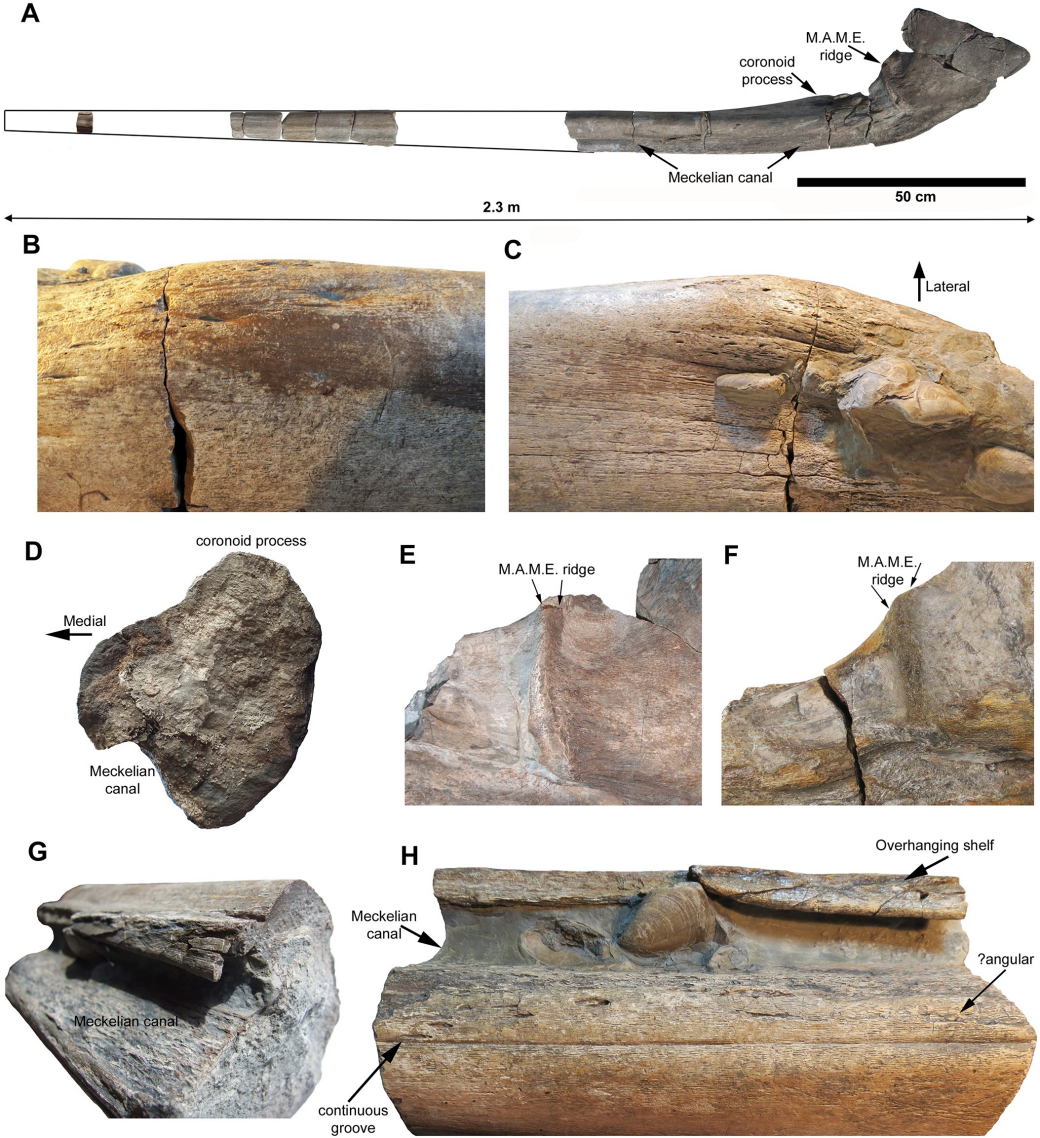
The holotype of *Ichthyotitan severnensis* gen. et sp. nov., a newly collected specimen (BRSMG Cg3178) comprising a very large, but incomplete right surangular (the ‘BAS Specimen’). A. All associated pieces with an approximate outline of the complete surangular, in medial view. The surangular is separated into two main parts, Part #A to the right and Part #B to the left (see text). B. A close-up of the coronoid process in lateral view, showing moderate eminence. C. Bulbous coronoid process in dorsal view with lateral displacement. D. Subcircular cross section at the level of the coronoid process (posterior view, medial to the left). E-F. Comparison of the massively developed M.A.M.E. ridge observed in BAS (E) and the Lilstock surangular (F); arrows indicate top of the ridge. G. Oblique view of the medial surface highlighting part of the overhanging shelf that encloses the Meckelian canal. H. Ventromedial view of the mid-posterior portion of the surangular showing a distinct, continuous, and straight thin groove that might be a suture and could indicate two distinct bones (perhaps including a damaged angular).

**Fig 3:**
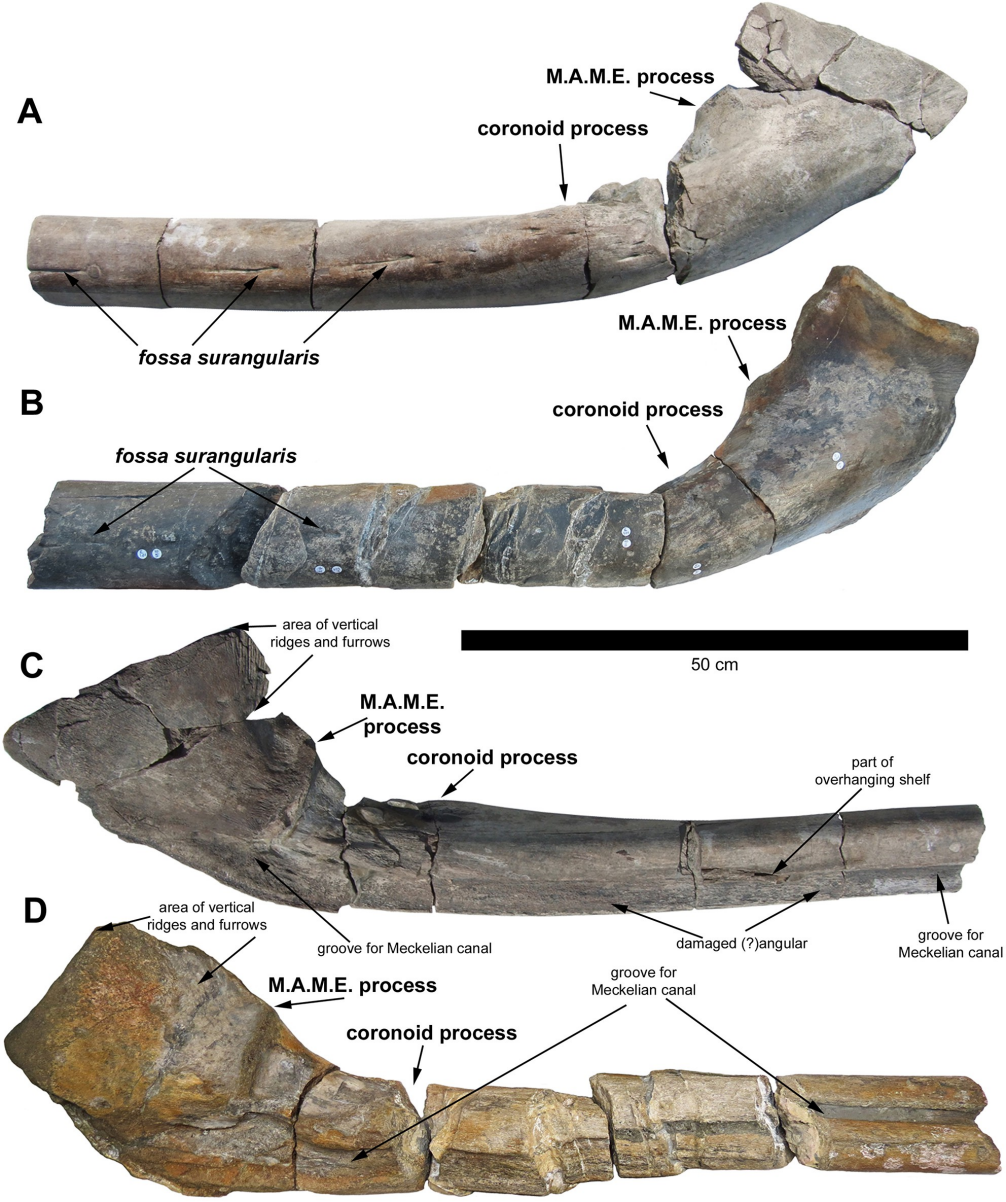
Comparison of the holotype (BRSMG Cg3178, A and C right surangular, BAS specimen) and referred specimen (BRSMG Cg2488, B and D left surangular, Lilstock specimen) of *Ichthyotitan severnensis* gen. et sp. nov. To ease comparison, A and C have been reversed. A-B. Lateral view of both surangulars showing same unique shape; note the upturned, almost 90-degree angle bend and the spatulate-shaped posterior end. C-D. Medial view of both surangulars displaying same morphology posteriorly; anteriorly the Lilstock specimen (D) has been heavily eroded and distorted along its length (see Discussion in Lomax et al. 2018 for more details). Note the position of an elongated foramen on the lateral surface (A-B), identified as part of the *fossa surangularis* that passes through the bone into the Meckelian canal. See also the damaged (?)angular that is articulated with the surangular and defined by a continuous groove (?suture) as seen in Fig 2H.

The newly found specimen that is the focus of this study, BRSMG Cg3178 (herein referred to as the BAS specimen, i.e. the Blue Anchor Surangular), is a large portion of an ichthyosaur surangular from the Westbury Mudstone Formation (latest Rhaetian), or Westbury Formation, collected at Blue Anchor, Somerset, UK (Figs 1–3; DOI: 10.6084/m9.figshare.25290661). A 3D photogrammetry model of the specimen can be found here, DOI: https://sketchfab.com/3d-models/ichthyotitan-severnensis-surangular-2d8556bc9cbd4cf1bd93b33044770a4f. BRSMG Cg2488, known as the Lilstock surangular, is also from the Westbury Mudstone Formation (latest Rhaetian), but was found at Lilstock, Somerset, UK (Figs 1 and 3; see supplementary data from [7] DOI: 10.6084/m9.figshare.5975440). As described by Lomax et al. [7], specimens BRSMG Cb3869, BRSMG Cb3870, and BRSMG Cb4063 are isolated, fragmentary bones from the jaws of giant ichthyosaurs collected from the ‘Rhaetic Bone Bed’ at the base of the Westbury Mudstone Formation (Rhaetian) of Aust Cliff, Gloucestershire, UK; specifically, BRSMG Cb3869 is probably a portion of a very large surangular. Comparisons are made with specimens of *Shonisaurus popularis* (those discussed in [3] and also figured in [7]), the holotype of *Shonisaurus sikanniensis* (TMP 1994.378.02, [5]), and the holotype of *Cymbospondylus youngorum* (LACM DI 157871, [1]). For further comparative purposes, we also examined MJML K2577, a comparable section of surangular belonging to an example of *Ophthalmosaurus icenicus* from the Oxford Clay Formation (163 Ma) of the upper Thames Valley, England. No permits were required for the described study, which complied with all relevant regulations. Whilst this manuscript was under review, the authors were made aware of a recent discovery of another large, albeit much smaller fragment of jaw from a giant ichthyosaur collected from Lilstock. The specimen is presently in a private collection, but it adds yet another example to the growing collection of giant Triassic ichthyosaurs in the UK. The specimen is not discussed further.

To inspect the bone histology of the new specimen (BRSMG Cg3178) and compare it with the unique bone histology described for the Aust bones [16] and for the Lilstock surangular ([7]; but see [17, 18]), we applied the core drill technique following Sander [19] and Stein and Sander [20] to produce histological samples to analyse. The core was extracted from the most anterior fragment of the posterior section of the surangular, in the immediate posterior portion of the clearly visible furrow (nutrient foramen), to match the sample area in BRSMG Cg2488 R-101 and BRSMG Cb3869 (see Fig 5). Two thin sections were produced from the core using wet silicon carbide powder of grit sizes 600 and 800 for grinding and polishing processes. Once covered, the thin sections were studied under a Leica DMLP light microscope with transmitted, cross-polarized light and circular polarized light. Circular polarized light [21] was obtained using a pair of commercially available 3D polarized lenses to replace the crossed polarizers. Photo-micrographs were obtained using a Mounted Dino-Eye camera (software DinoCapture 2.0 ver 1.5.45 © 2016 AnMo Electronics Corporation). Photo-micrographs were merged using Image Composite Editor (ver. 2.0.3 ©2015 Microsoft).

Histological terminology follows [22, 23] for general osteohistology. Networks of coarse collagenic fibres integrated in the periosteal bone matrix with no clear connection to external structures are defined as periosteal intrinsic fibres (PIF). PIF appear as a widespread structural character of the entire primary bone matrix scaffold not connected to external structures. A combination of PIF set in amorphous background matrix is defined as intrinsic fibre matrix (IFM). A woven-parallel complex with longitudinal osteons integrating IFM as a woven matrix, is defined as periosteal intrinsic fibre tissue (PIFT). The concepts of PIF, IFM, PIFT, and the concept of ‘template’ remodelling follow [18].

### Nomenclatural acts

The electronic edition of this article conforms to the requirements of the amended International Code of Zoological Nomenclature, and hence the new names contained herein are available under that Code from the electronic edition of this article. This published work and the nomenclatural acts it contains have been registered in ZooBank, the online registration system for the ICZN. The ZooBank LSIDs (Life Science Identifiers) can be resolved and the associated information viewed through any standard web browser by appending the LSID to the prefix http://zoobank.org/ [zoobank.org]. The LSID for this publication is: urn:lsid:zoobank.org:pub:D099EF35-0035-4520-A20E-806A1B8B4109. The electronic edition of this work was published in a journal with an ISSN, and has been archived and is available from the following digital repositories: PubMed Central, LOCKSS.

### Discovery and provenance

The BAS specimen (BRSMG Cg3178) was found in multiple pieces, including several parts of the better-preserved posterior portion found *in situ* (by JR, RR and PdlS). The first piece, representing a large, slightly worn bone section was collected on May 28, 2020, by JR and RR who were fossil collecting at the Blue Anchor location (Fig 1). An anonymous member of the public had left the bone on a large boulder at the top of the beach, presumably thinking that it might be something of interest. JR and RR searched the vicinity of the area and RR found a better preserved, larger section clearly showing the distinct Meckelian canal of an ichthyosaurian surangular. Together, both identified the material as bone and compared the finds with the surangular described by Lomax et al. [7]. As a result, they informed DRL and PdlS of the discovery. Several trips to the site led to the recovery of additional remains of BRSMG Cg3178, which were found by JR, RR, PdlS, DRL and others (see Acknowledgments).

The most recently collected piece of BRSMG Cg3178 was recovered by PdlS on October 16, 2022. Despite searching the foreshore and *in situ* location, no more material has come to light, although there remains the strong possibility that more of this individual is out there. As a result, the precise location of the BAS site is not being divulged at this stage due to the potential for more material to be found.

Additional remains collected on the foreshore include eroded pieces of bone, along with isolated rib fragments, vertebrae, and a phalanx all found loose on the beach in the vicinity of the area where BRSMG Cg3178 was found. It is not possible to confidently associate any of the material with the surangular. Two large rib sections were also found embedded in a mass mortality bivalve mollusc bed which is stratigraphically just above where the rest of the surangular pieces were found, which demonstrates these rib sections do not come from the same individual but another potentially large-bodied ichthyosaur. Additionally, a large coprolite containing fish scales was found in the vicinity. Unless stated otherwise, these additional elements are not discussed further.

**Fig 4:**
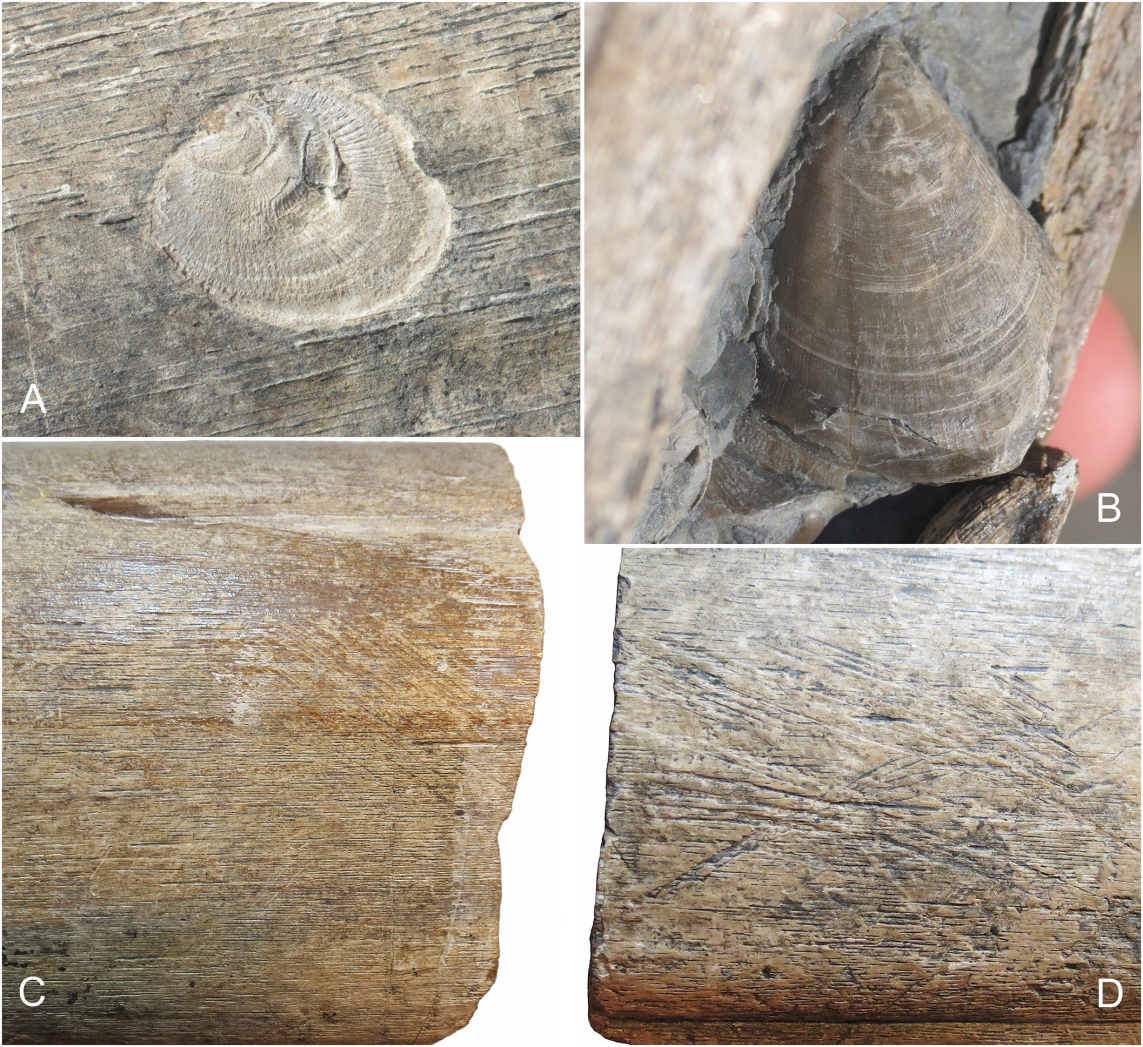
Invertebrate and trace fossils found on the bone surface of the BAS surangular, BRSMG Cg3178. A-B. Associated bivalves, including *Atreta intrusstriata* (A) and *Plagiostoma giganteum* (B); it is worth noting that a small group of the latter are preserved adjacent to the coronoid process, see Fig 2C. C-D. Examples of the probable scavenging marks that are also observed in the Lilstock surangular, see Lomax et al. 2018, Fig 4.

Similar to the left surangular described by Lomax et al. [7], the BAS specimen comes from the highest part of the Westbury Mudstone Formation (Upper Triassic, Rhaetian) and was found in twelve distinct pieces (Fig 2A). Like the Lilstock surangular, the specimen shows signs of abrasion and encrusting organisms, including multiple bivalves (*Atreta intrusstriata* and *Plagiostoma giganteum*, DRL pers. comm. Crispin Little 2023) and probable scavenging/scratch-like marks (see also [7] Fig 4; Fig 4). Considering the stratigraphical position of this specimen, as in the Lilstock surangular, it narrowly predates the Triassic-Jurassic extinction event found in the above Cotham Formation ([7, 24, 25]). Together, these specimens represent the latest Triassic occurrence of giant ichthyosaurs in the UK.

### Systematic palaeontology

Ichthyopterygia Owen, 1840

Ichthyosauria de Blainville, 1835

?Merriamosauria Motani, 1999

?Shastasauridae Merriam, 1902

*Ichthyotitan severnensis* gen. et sp. nov.

LSID for the genus: urn:lsid:zoobank.org:act:57B85E76-0A91-4EBF-9634-5B5A5FB10B60

LSID for the species: urn:lsid:zoobank.org:act:53F19051-D7E5-4ADB-8129-48D66B79C7A2

#### Etymology

Giant fish lizard of the Severn. *Ichthys* derived from Greek meaning fish, taken from ichthyosaur meaning “fish lizard”, and -*titan* (Greek for giant), after the large size. Severn after the River Severn Estuary, Somerset, UK, where the remains were discovered and Latin -*ensis* pertaining to the location.

#### Holotype

BRSMG Cg3178, a large right surangular comprising the posterior end and parts of the middle and anterior sections.

#### Referred material

BRSMG Cg2488, a large left surangular comprising the posterior end and a portion of the shaft.

#### Type locality and horizon

The type specimen was collected from the Upper Triassic Westbury Mudstone Formation (latest Rhaetian) at Blue Anchor, Somerset, UK. The referred specimen was collected from the Upper Triassic Westbury Mudstone Formation (latest Rhaetian) 0.8 m below the junction with the Cotham Formation, at Lilstock, Somerset, UK.

#### Diagnosis

Giant, probable shastasaurid ichthyosaur distinguished by the presence of the following unique characters of the surangular: upturned, almost 90 degree angle bend at posterior end; subcircular cross section morphology of the shaft at the position of the coronoid, oblong in *Shonisaurus*; minor eminence of coronoid process in lateral view, compared with prominent projection in *Shonisaurus*; bulbous coronoid process displaced laterally and only occupying half of the width of the dorsal surface; massively developed dorsoventral M.A.M.E. ridge; spatulate shaped posterior end; and possibly dorsoventral height of posterior end in adults being more than 20% larger than in either *Shonisaurus popularis* or *Shonisaurus sikanniensis*.

#### Remarks

Further to the last character in the diagnosis, this feature can be used to distinguish the new taxon from other giant ichthyosaurs, as mentioned, but this could be problematic for assigning immature or juvenile individuals of *I*. *severnensis* that would be smaller than adult specimens of *Shonisaurus* spp. Therefore, we feel that this character can be used to compare large, presumably adult specimens but might not be useful (unless scaling is taken into consideration) in assigning smaller individuals that may be discovered in the future. Another notable feature that might be of interest is a continuous, straight thin groove in the ventral surface of the BAS specimen. For further details, see the description below. Probable shastasaurid affinities are based on the large size of the ichthyosaur combined with the Upper Triassic age, a time when the largest-known ichthyosaurs (the Shastasauridae) existed.

### Description and analysis

The holotype of *Ichthyotitan severnensis* (BRSMG Cg3178, BAS specimen) is a large, robust but incomplete right surangular, uncrushed and preserved in three dimensions (Figs 2, 3A and 3C). The only referred material comprises the Lilstock specimen (BRSMG Cg2488), which represents a near identical albeit less complete left surangular, preserved in three dimensions with a length of 96 cm (Fig 3B and 3D). The BAS specimen was chosen as the holotype because it is more complete and is generally better preserved. The Lilstock surangular was described in detail by Lomax et al. [7] and is, where appropriate, compared here with the new specimen. Relatively few surangulars are known from the largest Triassic ichthyosaurs and uncrushed three-dimensional preservation of isolated ones is rare. This is partly the reason why such bones have previously been misidentified [7].

The holotype is separated into at least 12 distinct sections that are associated and split into two main portions, termed Part #A and Part #B to aid with description (Fig 2A). The best-preserved part of the surangular is the middle to posterior portion (Part #A), made up of five key segments, which is approximately 99 cm in length. A more anterior section (Part #B) of the surangular was found scattered in five pieces on the foreshore and shows a moderate amount of wear from erosion. This section measures 46 cm long. The connecting material between these two sections of the surangular, along with the anterior-most portion, have not been found. Two additional pieces of the anterior portion were collected but the position of only one of them could be approximately located on the surangular (see Fig 2A). We can be confident of the association between the anterior and posterior portions due to them being found in the immediate vicinity of each other, along with their size and their shared morphology. The combined preserved length of Part #A and Part #B measures 145 cm. Based on the extrapolation of measurements of the tapering thickness of the anterior portion, which indicates a potential minimum gap between the two main sections of 38 cm (Fig 2A), combined with the additional piece from the anterior region suggests a significant portion of the anterior is missing. We estimate the total length of the surangular to be >2 m (Fig 2A).

As in the Lilstock specimen, the posterior end of the BAS surangular is thick and dorsoventrally tall with an almost 90-degree curve (Figs 2A and 3). Lomax et al. [7] considered that the curvature might be the result of taphonomic distortion in the Lilstock specimen, but the preservation in BAS is much better and confirms that the curvature is natural. Nevertheless, when compared side-by-side in medial view, the Lilstock surangular is clearly distorted at the posterior-most end where the straightened edge of the dorsal surface is rotated posteriorly, a feature that is correctly oriented and in a natural position in the BAS specimen (compare Fig 3C and 3D). As discussed in [7], a similar but less marked curvature occurs in the surangular of *Shonisaurus sikanniensis* and a slight curve is present in *S*. *popularis*. Lomax et al. [7] further noted that there might be some differences in the degree of curvature among taxa, stating that it is impossible to determine the significance with such a small sample size. However, we now have both the Lilstock and BAS specimens with the same degree of dorsoventral curvature. Overall, the rear section of the BAS surangular is remarkably similar in size and morphology to the Lilstock specimen. The maximum dorsoventral height is at the posterior end of the surangular and is 26 cm, as preserved.

A small, bulbous coronoid process with minor eminence is present and is laterally directed and laterally displaced, making it difficult to observe in lateral view (Figs 2B, 2C, 3A and 3C); this morphology differs greatly from *Shonisarus popularis* which has a prominent triangular coronoid process (compare Fig 2A with [7], Fig 7A). It only occupies half of the width of the dorsal surface in the coronoid region and the medial side shows a slight concavity in this region (Fig 2C). Its surface is pitted with numerous forward-directed foramina and muscle scars. Note that due to distortion present in the Lilstock specimen, the identification observed herein on the BAS specimen shows that the coronoid process was misidentified in Lomax et al. [7] leading to the incorrect placement of the M.A.M.E. (M. adductor mandibulae externus) process in the Lilstock surangular (Fig 3). Slightly anterior to the coronoid process, this part of the bone is subcircular in cross section, unlike in the surangular of *Shonisarus popularis*, which is distinctly oblong in cross section (see Fig 19 in [3]). As a result of the robustness at the point of the coronoid process, this section of the surangular is wider than it is anteriorly (Fig 2D).

There is a minor reduction in the dorsoventral height immediately posterior to the coronoid process. However, the height then greatly increases dorsally at the point of the almost 90-degree curve, marked by the presence of a massive, prominent and extensive M.A.M.E. ridge on the medial side for muscle attachment (Fig 3). The ridge shows clear muscle attachment scars, as in the Lilstock specimen. A thin process curves dorsally from a point posterior to the M.A.M.E. at an angle of 90 degrees to the main shaft of the bone. This process carries prominent vertical ridges and furrows on its medial side (Figs 2E, 2F, 3C and 3D), as reported in the Cuers ichthyosaur [26]. The purpose of this feature is unknown.

The Meckelian canal is prominently visible along the medial side of both parts (Part #A and #B; Figs 2A, 3C) and would have provided a conduit for the passage of nerves and blood vessels (and cartilage during bone growth). It is partially filled with a light grey matrix and preserves bivalves in some places (Figs 2H and 4B). The distalmost part of the posterior end of the ramus narrows laterally and expands dorsoventrally forming a large, spatula-shaped concavity for the reception of the articular bone (not present). Towards the anterior-mid section of Part #A, there is evidence of a broken, overhanging shelf enclosing the Meckelian canal dorsally along most of its length, from the anterior to the region of the coronoid process. In one area this is complete (Figs 2G, 2H and 3C) and is likely for the reception of the dentary. In the posterior region, the Meckelian canal opens up posteriorly into a large fossa pierced by a number of foramina, the largest of which connects through the bone to the *fossa surangularis* on the lateral side (Fig 3). The *fossa surangularis* opens into a number of forward-directed, elongated foramina on the lateral side of the rear section of the bone and into a continuous fossa along the lateral side of the anterior section (Figs 2A and 3A); this fossa is visible in the bone cross sections. It is also observed in the Lilstock specimen and another large section of probable surangular (BRSMG Cb3869), where those bones also bear multiple, fine longitudinal indentations, preserved across the lateral and dorsal surfaces.

As described above, the posterior part of the surangular (Part #A) is the better preserved of the two main portions, showing little erosion or distortion. However, the anterior part of the surangular (Part #B) is more complex in cross-sectional outline than the posterior, showing complex concave facets on both the medial and lateral surfaces. Presumably, these are for the reception of the dentary on the lateral side, and the splenial on the medial side. The large lateral groove on this anterior portion probably represents the *fossa surangularis* in this region. This feature is also seen in the Cuers ichthyosaur ([26] Fig 2), a large incomplete surangular [see 7].

A damaged portion of bone is present ventral to the Meckelian canal, best viewed along the lower medial side (see e.g. Fig 2H). Through direct comparison with the holotype of the Early Triassic *Cymbospondylus youngorum* (DRL and JFW pers. obs.), for which a well-preserved and articulated skull exists, the position of the bone suggests that it is probably part of the angular. It is present along the entire length of the surangular and can be seen on both the anterior and posterior sections, although best preserved on the posterior part (Part #A). There is a distinct, continuous, and straight thin groove (a probable suture) visible between the two bones at the surface. However, a close examination of the internal bone structure, visible in cross section, shows that it is continuous across the bone junction, with no discontinuity in cell structure and no intervening matrix. This may indicate that the bones were possibly fused together in life with no fossa separating them. If so, this would be unusual and possibly unique among ichthyosaurs, perhaps related to the large size and presumed mature nature of the individual. On a section of bone just anterior to the coronoid process, a vestigial suture between the surangular and (?)angular can be seen to disappear completely, further confirming the apparent fusion. A similar morphology is present in at least one of the Aust bones (BRSMG Cb3869), as also alluded to by Huene [27]. The Lilstock surangular is poorly preserved in ventral view, but direct observations suggest that there might also be a faint groove.

### Results of core sampling

**Fig 5:**
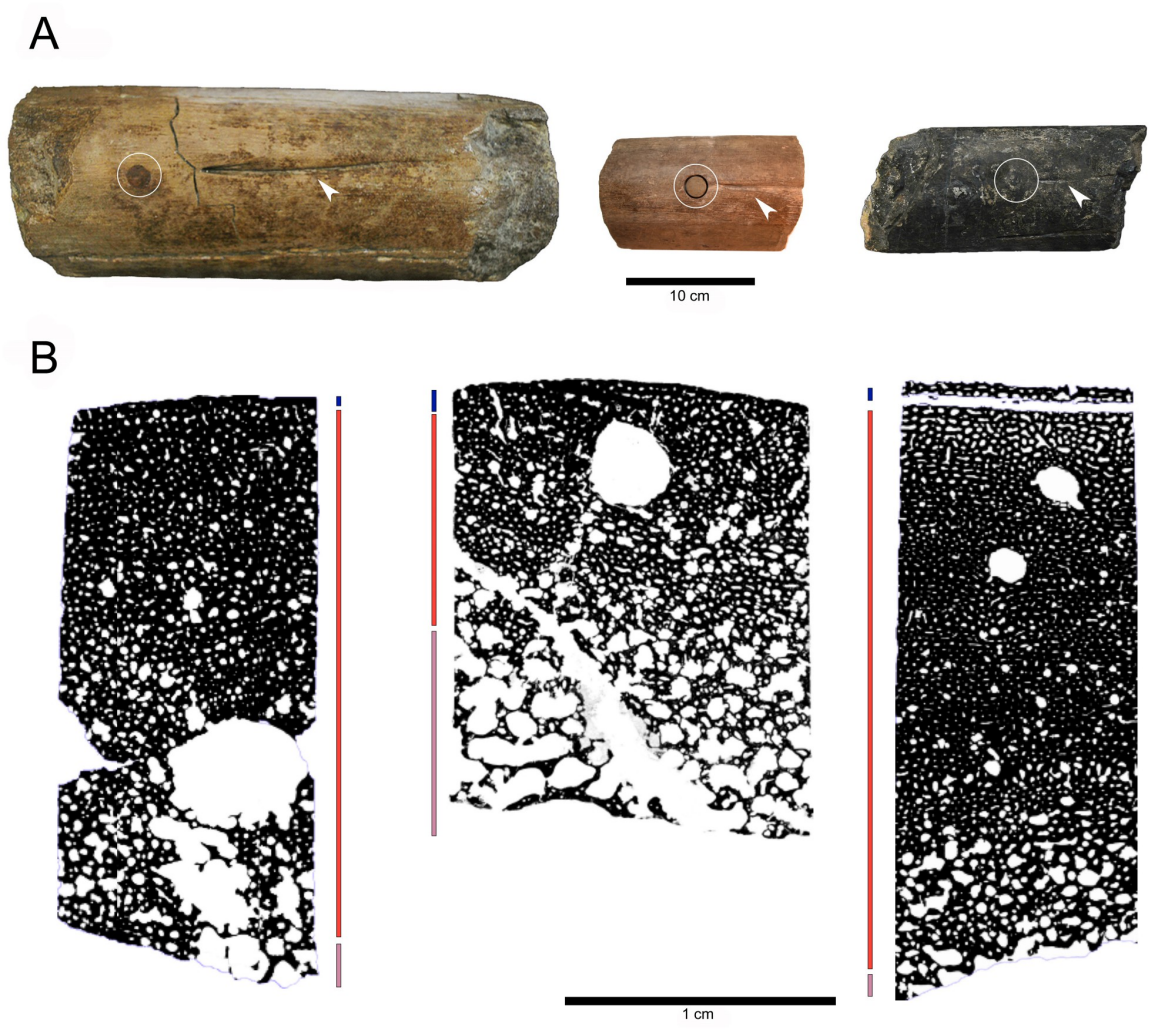
A. Comparable sections for core drill(s) sampling position indicated by a white circle of (from left to right) BRSMG-Cb-3869 (an Aust bone, most probably a surangular), BRSMG Cg3178 (BAS surangular), BRSMG-Cg-2488 R-101 (Lilstock surangular). White arrows point to elongated surangular foramen. B. Binary drawings produced from stitched photos of the thin sections (respectively BRSMG-Cb-3869, BRSMG Cg3178 and BRSMG-Cg-2488 R-101) showing longitudinal vascularization and larger nutrient canals. Blue bars (upper) indicate extension of outer cortex, orange (middle) for deep cortex and pink (lower) for spongious trabecular bone.

**Fig 6:**
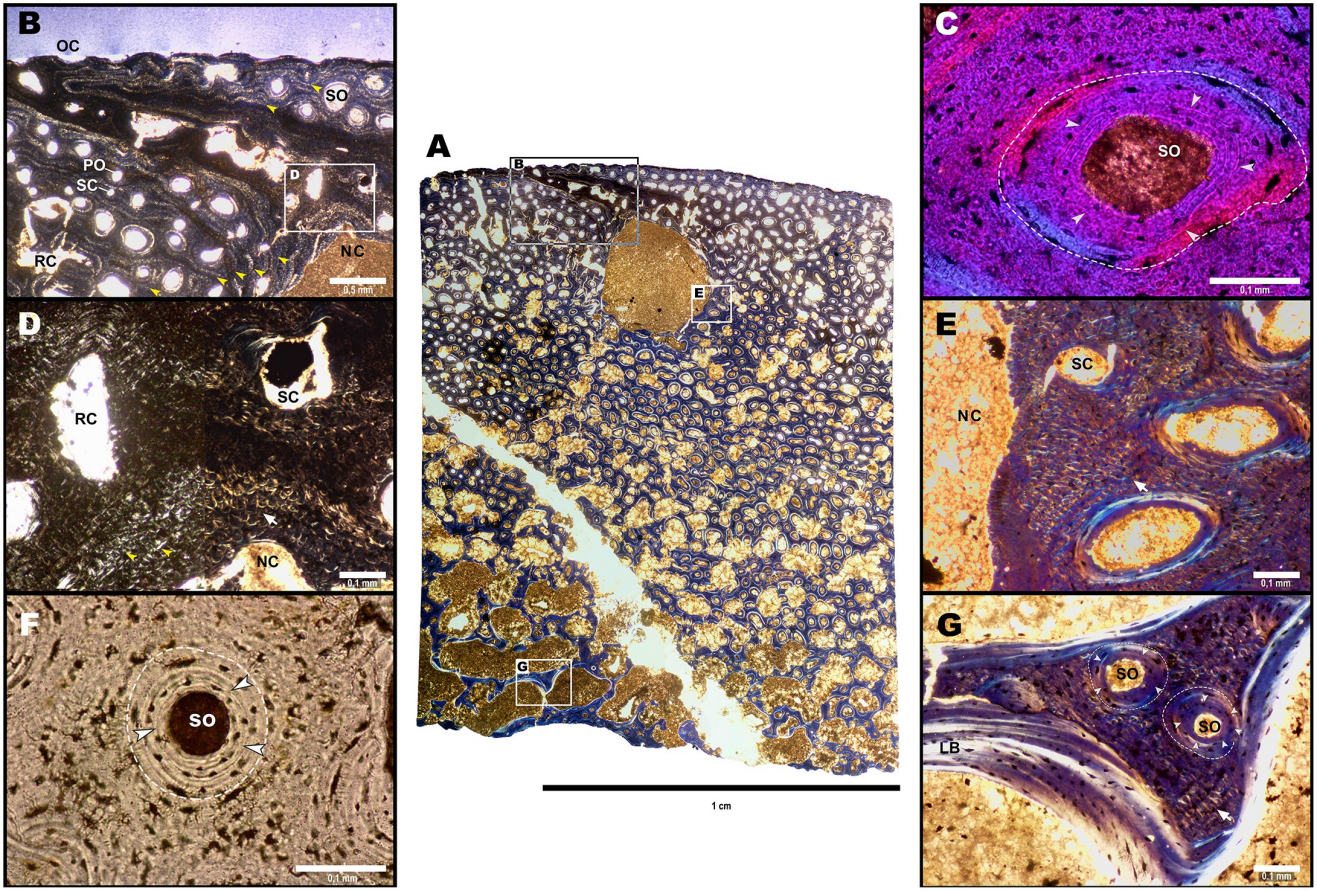
Histological overview of BRSMG Cg3178 (BAS surangular). A. Composite image of thin section under circular polarized light. B. Close-up of the external margin of the outer cortex, showing the presence of multiple growth marks (GMs), open vascular canals and cortical vascular canals with all degrees of maturity (simple canals, primary osteons and secondary osteons), supporting an ongoing active and continuous growth. Note the evident darker border of the lumen of a diagonal canal running from the top left toward the margin of the large nutrient canal (NC) showing further longitudinal vascularization. C. Concentric secondary osteon in the outer cortex under lambda filter. D. Close-up of the upper margin of the nutrient canal under crossed polarized (left) and circular polarized light (right). The growth marks appear as alternated tightly packed rows of brighter and darker periosteal intrinsic fibres (PIF). The same tight packing of the GMs occurs also deeper in the cortex. E. Lateral margin of the nutrient canal under circular polarized view. PIF are evident as bright yellow and blue coiled structures. The presence of simple canals alongside osteons, indicates primary deposition of bone along the margin of the large nutrient canal. F. Concentric secondary osteon in the trabecular bone under transmitted light. It is evident the high amount of osteocyte lacunae and the presence of plump irregular shaped ones in the lamellar bone. G. Trabecular bone under circular polarized view. The presence of primary matrix and concentric secondary osteons indicate that the trabeculae are secondary, produced from compact bone made cancellous. White arrows (D, E, G) point at PIF; white arrowheads point at resorption lines in concentric osteons (C, F, G); white dotted lines indicate borders of primary osteons (C, F, G); yellow arrow heads (B, D) point at rows of GMs. Abbreviations. LB, Lamellar bone; NC, Nutrient canal; OC, Open periosteal canal; PO, Primary osteon; RC, Resorption cavity; SC, Simple canal; SO, Secondary osteon.

The section taken from BRSMG Cg3178, the BAS specimen (Fig 5A and 5B middle), shows good preservation, although multiple osteons showing irregular breakage rims without clear Howship’s lacunae, and a long diagonal fracture in its lower half (Figs 5B and 6A) indicate diagenetic or taphonomic-related damage. The bone histology is characterized by a highly vascularized woven-parallel complex [22, 23] (Figs 5B, 6A and 6B). Vascular canals are strictly longitudinally oriented (Figs 5B and 6A). The primary bone matrix is characterized by a complex of networks of intrinsic longitudinal mineralized collagen fibres (periosteal intrinsic fibres, or PIF [18]) (Fig 6D, 6E and 6G). Under cross-polarized light, PIF appear bright against an isotropic amorphous dark background matrix (Fig 6D). PIF appear as circular or spirally coiled structures under circularly polarized view (Fig 6D, 6E and 6G). The network of PIF set in an amorphous dark matrix (Fig 6D) defines an intrinsic fibre matrix, IFM, [18] (and see Materials and methods). IFM is identifiable both in the trabeculae interstitial bone (Fig 6G) and, as primary matrix of the rest of the cortex (Fig 6D and 6E). The section can be subdivided into an innermost half of spongious trabecular bone (trabecular region), and a compact cortex that can be further subdivided for description purposes, into a deep cortex and an outer cortex (Fig 5B).

The trabecular region presents secondary trabeculae constituted by compact bone made cancellous (sensu [22]) (Figs 5B, 6A and 6G). The trabeculae are composed by islands of IFM bordered by large resorption cavities (RC) and hemiosteons (Fig 6A and 6G). RC and hemiosteons are lined by relatively few layers of lamellar bone (Fig 6G). It is possible to observe a preferential orientation of the trabecular bone in the horizontal plane (Figs 5B and 6A).

Passing from the trabecular region to the deep cortex, the size of the RC gradually diminishes (Figs 5B and 6A), giving space to a highly vascularized, well-arranged compact bone showing large numbers of secondary osteons that generally appear in an in-row arrangement (Fig 6A). The in-row arrangement appears parallel or slightly subparallel to the subperiosteal outer surface. The primary tissue constituting the compact cortex is periosteal intrinsic fibre tissue (PIFT) observed by Perillo and Sander [18].

A close inspection following the methods described in [16, 18] shows that secondary osteons present successive resorption lines inside the osteon lamellar infill (Fig 6C and 6F). This phenomenon results from a consistent spatial correspondence between basal metabolic unit development and previously existing vascular canals. Secondary osteons develop preferentially inside preexisting osteons, either primary or secondary (in the latter case these are named concentric osteons). This phenomenon defines a ‘template’ remodelling [18]. It is nonetheless possible to see many resorption cavities in the deep cortex, although some of this may be related to preparation damages due to their shape. The number of resorption cavities present in the deep cortex increases the overall porosity of the bone structure (Figs 5B and 6A) and suggests an advanced resorption front [28].

In the deep cortex, going toward the subperiosteal surface, the number of primary osteons, immature secondary osteons and concentric secondary osteons increases, while the amount of resorption cavities and non-concentric osteons decreases. Compared to the cancellous bone, the compact cortex shows also an increased amount of PIF clearly visible (Fig 6B and 6E). In the upper deep cortex and outer cortex, it is possible to identify numerous (~15) closely positioned wavey GMs formed by the alternation of brighter and darker layers of PIF (Fig 6B and 6D). The width and colour of the GMs is variable with darker and brighter ones alternating with no clear pattern. These GMs embrace the primary osteon and vascular canals and originate from periosteal apposition but do not show changes in tissue type, being always composed of IFM. Given the absence of a change of tissue type, these GMs are not identifiable as LAGs (see Discussion). By close inspection, a scarce number of similar GMs, are identifiable in the rest of the deep cortex, always embracing the parallel rows of osteons but showing less continuity due to the higher number of RC.

A large longitudinal resorption cavity (diameter around 0.75 mm, area around 2 mm^2^) occupies the upper central area of the section in the deep cortex (Figs 5B and 6A), and is identifiable as a nutrient canal. The rim of the canals shows primary IFM (Fig 6E), primary osteons and simple canals, as well as Howship’s lacunae, indicating that this area was actively subjected to osteogenic and osteoclastic processes through the developmental stages of the animal. The large nutrient canal is connected to the outer surface by a large diagonal canal (Fig 6A and 6B). The section of the canal lumen measuring 0.3 mm in diameter (but tapering to 0.15 mm in its middle, assuming an hourglass shape). The diagonal canal is detectable by the darker colour of its lumen wall compared to the surrounding bone (Fig 6A and 6B). The borders of the diagonal canal show a clear continuity with the surrounding periosteal rims of the outer cortex as suggested by the organization of osteons and GMs (Fig 6B). The lumen wall of the diagonal canal shows longitudinal simple canals and secondary osteons orderly arranged parallel to its diagonal orientation (Fig 6B). Both under cross polarized view and circularly polarized view the canal lumen wall shows the presence of PIF (Fig 6B and 6D), indicating it is made of IFM.

In correspondence with the border of the large nutrient canal, the GMs show a clear descending down turning (Fig 6B). This skewed arrangement indicates that the periosteal apposition process was influenced by the presence of the major nerve and/or blood vessel occupying the nutrient canal.

The outer cortex shows simple canals, primary osteons and incipient concentric secondary osteons (Fig 6B and 6D). It is not possible to identify an External Fundamental System. The subperiosteal surface is characterized by various open periosteal canals (Figs 5B, 6A and 6B). The latter show rims with no signs of breakage, therefore are identifiable as genuine biological open canals. The occurrence of open periosteal canals and simple canals indicates active growth at the time of death, as for some of the Aust bones described by [16]; see also [7]. The borders of the nutrient canal show primary bone tissue with simple vascular canals and primary osteons, resembling the condition observed on the outer cortex (Fig 5B, 5D and 5E). Numerous osteocyte lacunae are evident in the entirety of the section: dynamic flattened osteocytes are commonly found in lamellar bone (Fig 5C, 5E and 5G), while plump or irregular shaped ones are found both in the PIFT matrix and in its lamellar bone (Fig 5F and 5C).

### A note on size estimations

When describing the Lilstock surangular, Lomax et al. [7] used large shastasaurid ichthyosaurs to provide a rough total length estimate of the ichthyosaur and for one of the Aust bones (BRSMG Cb3869), but emphasised the need for caution with such estimates when dealing with isolated remains. To determine these size estimates, using a simple scaling factor, they compared (1) the maximum dorsoventral height at the posterior end of the Lilstock surangular with the same point in the surangular of the large *Shonisaurus sikanniensis* and (2) the height of the Lilstock surangular at the coronoid process compared with the same in a smaller shastasaurid, *Besanosaurus leptorhynchus* [29]. Based on these comparisons, Lomax et al. [7] found that the Lilstock ichthyosaur had an estimated total length of between 26 and 22 m respectively and further stated that it is reasonable to suggest that the Lilstock ichthyosaur was on the order of 20–25 m long.

However, being better preserved than the Lilstock specimen, the BAS surangular has revealed that the identification of the position of the coronoid process and M.A.M.E. process on the Lilstock surangular are incorrect (see Fig 3) and therefore the size estimate compared with *B*. *leptorhynchus* is inaccurate. The position used by [7] for the measurement of the coronoid process (2) in the Lilstock surangular is actually the position of the M.A.M.E. Therefore, we use the position of the dorsoventral height of the M.A.M.E. in BAS, which measures 19 cm, and is the same height as that previously identified as the coronoid (now M.A.M.E.) in Lilstock, compared with the same point in *B*. *leptorhynchus* that measures approximately 3.5–4 cm at the M.A.M.E. (based on the illustration by Bindellini et al., [30], Fig 4; see also [7]). Note, however, that [30] follow a different terminology and refer to the M.A.M.E. in *B*. *leptorhynchus* as the coronoid process, but we regard their coronoid process as the M.A.M.E.; nevertheless, the measurement and scaling remains the same regardless of the identification. Based on this revised comparison, it suggests that the Lilstock/BAS ichthyosaurs are almost five times larger than *Besanosaurus* (which has a total body length of 5.4 m) with the largest total length estimate of 25 m, thus still in the 20–25 m range given by Lomax et al. [7].

**Fig 7:**
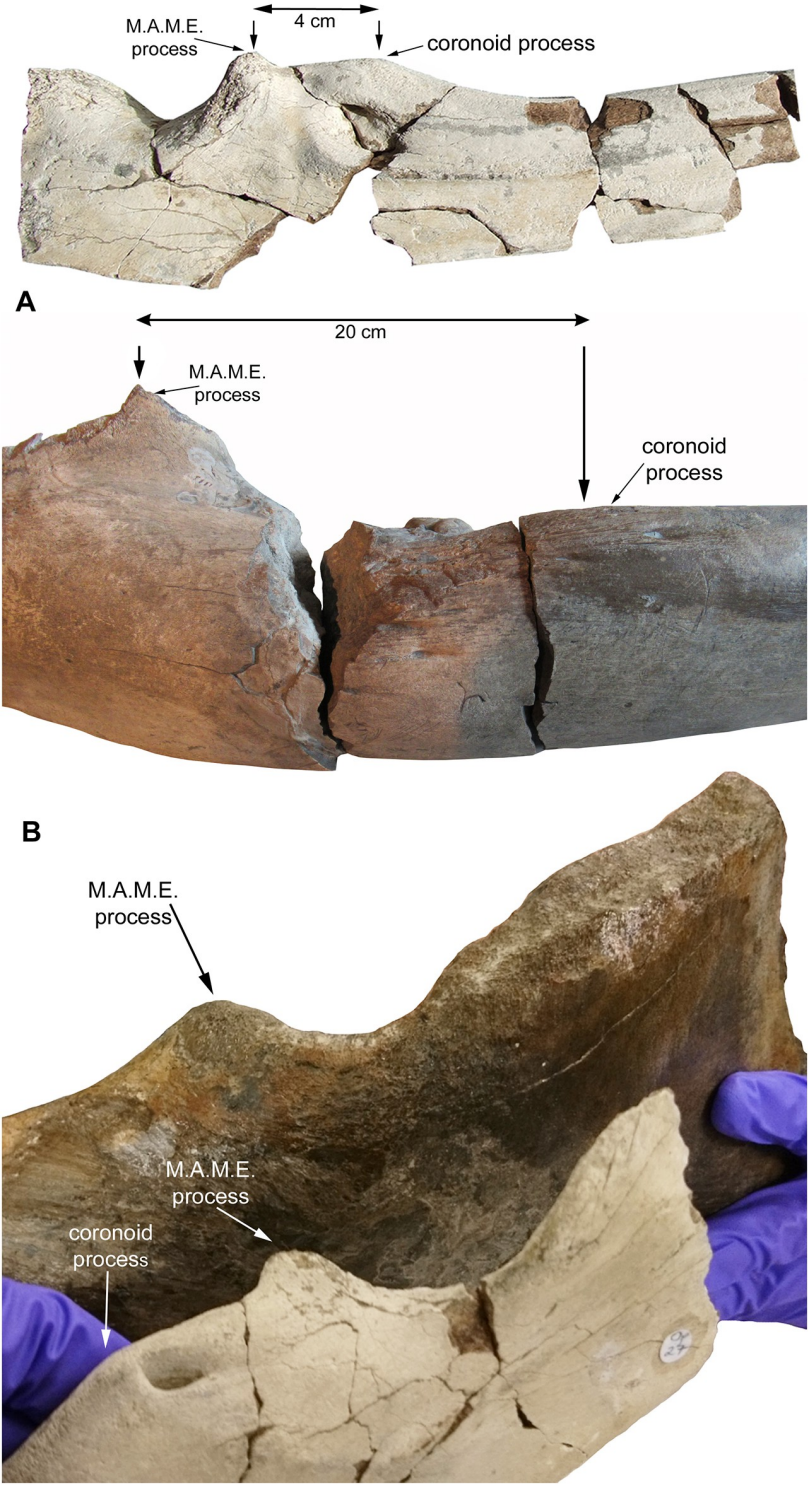
Surangular comparisons between the holotype (BRSMG Cg3178, BAS specimen) and referred specimen (BRSMG Cg2488, Lilstock specimen) of *Ichthyotitan severnensis* gen. et sp. nov., with a comparable section of surangular from a specimen of *Ophthalmosaurus icenicus* (MJML K2577). A. BRSMG Cg3178 and MJML K2577 illustrating the distance between the M.A.M.E. and coronoid process. B. BRSMG Cg2488 and MJML K2577 are positioned obliquely in lateral view (with MJML K2577 rotated and held closer to the camera), illustrating the general shape of the ichthyosaurian surangular.

To test the simple scaling further, we also compared the BAS specimen with a posterior section of surangular from an example of the common Upper Jurassic ophthalmosaurid ichthyosaur, *Ophthalmosaurus icenicus* (MJML K2577; Fig 7). This specimen was chosen due to the same portion of surangular being three-dimensionally preserved and the fact that the M.A.M.E. and coronoid processes can easily be identified and compared between both specimens (see Fig 7). As a result, we could provide a new scaling comparison. This time, by comparing the distance between the M.A.M.E. and coronoid processes in the *Ophthalmosaurus* specimen (measuring 4 cm) and the same in BAS (measuring 20 cm), this suggests an individual with an estimated body length five times greater, at 25 m long, the same estimate as presented above.

It is also worth reiterating the point made by Lomax et al. [7] that if the Aust specimen BRSMG Cb3869 is part of a surangular, which appears correct (see Fig 5A), then by using the same simple scaling this individual would be estimated at a very speculative 30+ m. Based on the analysis of the BRSMG Cb3869 bone microstructure [16, 18], this section is from an animal that was still growing at the time of death [16, 18] and might be due to prolonged growth strategies as previously suggested (see Discussion in [7]).

## Discussion

Confidently assigning isolated, fragmentary, or poorly preserved remains to a specific taxon is challenging and can be problematic when key features are lacking, or insufficient material is preserved. When describing the Lilstock surangular, Lomax et al. [7] considered that the specimen might have possible shastasaurid affinities based on its geologic age and giant size. They further showed that it had an unusual morphology that appeared unique but refrained from assigning a specific name to the specimen due to its incompleteness, taphonomic distortion and the fact that it was represented by an isolated bone. Moreover, surangulars from the largest Triassic ichthyosaurs are poorly known and any three-dimensional specimens are rare, therefore making direct comparisons difficult. However, those specimens that could be compared were found to differ from the Lilstock specimen [7].

The discovery of yet another surangular (BRSMG Cg3178) possessing the same morphology as the Lilstock specimen, but which is much better preserved and more complete, provides additional support for the identification of something unique. Moreover, it illustrates that the Lilstock specimen was not simply a single bone with an unusual morphology. Although we appreciate that this new taxon is based on two large incomplete surangulars, we feel that having two identical bones showing the same unique morphology, and which were collected from the same geological formation separated by ~10 km, is enough to warrant the erection of a new taxon. Furthermore, these specimens are approximately 202 million years old, from the late Rhaetian, and appear roughly 13 million years after the stratigraphically latest giant Triassic ichthyosaurs with a name, *Shonisaurus sikanniensis* from British Columbia, Canada [5], and *Himalayasaurus tibetensis* from Tibet, China [31]. Thus, given the differences in surangular morphology compared with *Shonisaurus* (e.g. compare the surangular morphology with *S*. *popularis* figured in [7], Fig 7a) along with the stratigraphic and geographic separation, it is highly unlikely that *Ichthyotitan severnensis* is an example of *Shonisaurus* or *Himalayasaurus*, which would also suggest an extremely long stratigraphic record and a much wider palaeogeographical distribution than what is known for both genera. Having two examples of the same bone with the same unique features from the same stratigraphic time zone supports the erection of a new taxon.

Considering the Rhaetian age and similar morphology of the bones, an argument could perhaps be made for the Aust bones (at least BRSMG Cb3869) and the Cuers ichthyosaur, a large surangular from the Rhaetian of France [26]; identified as a surangular by [7], to represent additional examples of *I*. *severnensis*. However, given that the Aust bones are stratigraphically slightly older (see [7], Fig 3), are much less complete and therefore do not show all the diagnostic features found in both the Lilstock and BAS specimens, we do not assign them to this species in this study. Similarly, although the Cuers ichthyosaur is more complete and shows some similarities with both specimens (see [26], Fig 2), the posterior end is somewhat poorly preserved and does not show the defining features necessary for positive identification. Nonetheless, these findings demonstrate that giant Rhaetian ichthyosaurs were present in the Tethys during this time. Similarly, it is worth noting that a fragmentary ichthyosaur comprising 17 ribs and two vertebral centra was recently discovered in the Gabbs Valley Range of Nevada, USA. Although this specimen has yet to be formally described, it demonstrates that giant ichthyosaurs were also present in Panthalassa during the Rhaetian [32].

Based on the core sampling results, the thin sections confirm that the BAS specimen shares peculiar histological characters with sections produced by other giant ichthyosaur specimens 16–18]. The same unique histology of the bones is supported by the presence of: 1) PIFT, 2) GMs determined by different arrangement e/o density of layers of PIF, 3) strictly longitudinal vascularization, 4) concentric osteons determining a cortical ‘template remodelling’. Thus, the results of this study are in agreement with the conclusions reached previously [16–18]. Furthermore, even in its absence, we predict that longitudinal sections of this specimen would most likely show the same structural collagen fibres arranged in a herringbone pattern, as observed in other giant ichthyosaurs [17, 18]. The presence of a nutrient foramen in the BAS specimen, as with similar structures observed in both BRSMG Cg2488 R-101 and BRSMG Cb3869, further supports the anatomical homology hypothesized for these specimens [7, 17,18]. Finally, it is noticeable that the horizontally oriented trabeculae, and the ratio of trabecular bone to compact cortex are also reminiscent of what was observed in BRSMG-Cb-3870 [16–18]. Although the consequences of matching histology paired with similar morphology of these specimens are intriguing, the lack of a more refined taxonomic resolution of the fragmentary material prevents support to any stable conclusion regarding the phylogenetic attribution to *Ichthyotitan*.

In the context of understanding the growth patterns and the developmental stage of BRSMG Cg3178, it is relevant to discuss the nature of the GMs we observed. The closely spaced GMs are, at first glance, reminiscent of LAGs forming the external fundamental system (EFS), which would advocate for an adult state of the animal and for the reaching of a stoppage in growth (i.e. [33]). Due to the same similarity, the identification of LAGs and the occurrence of an EFS was proposed by Redelstorff et al. [16] for another similar specimen (BRSMG-Cb-3870). It is noticeable, however, that the same study highlighted the thick width of BRSMG-Cb-3870 EFS as unusual and offered an alternative hypothesis to their conclusions. The GMs described here are characterized by a combination of higher brightness, lower brightness, and colour change of the same primary bone (IFM) and show no clear tissue type change (no lamellar bone or parallel fibered bone). This sharply contrasts with what defines LAGs [22], as already supported by [18], which described the same GMs from the specimens studied therein. The GMs described here are structures derived by modulations of orientation and density of longitudinal coarse fibres (PIF) deposited by active periosteal apposition [18] but with no clear connection to slowdown in growth. The identification of GMs as simple marks derived by changes in density and orientation of coarse longitudinal fibres is not unheard of, although it was observed in metaplastic tissue [34, Fig 2e and 2f]. To conclude, we do not observe an EFS given the absence in the outermost cortex of few layers of tightly packed LAGs. The absence of an EFS supports that the animal had yet to reach an asymptotic growth stage. The occurrence of the GMs already in the upper deep cortex, with no appreciable trend toward a diminishing space within them in the outer cortex, can be used as a further argument to exclude an ongoing progressive reduction in growth rate.

Further support for ongoing growth is offered by outer cortex vascularization. In both BRSMG-Cb-3870 [16, 18] and our specimen, as well as in BRSMG Cg2488 R-101 [18] and BRSMG Cb3869 [16, 18], the occurrence of multiple open periosteal canals and high vascularization in the outer cortex indicate that growth by periosteal apposition was not ceased at the moment of death. The few histological analysis carried on ichthyosaur rostra found them to be more compact than postcranial material [35]. We can therefore support that the high vascularization is a genuine indicator of active growth rather than being related to an increased osteoporotic specialization of the lower jaw. The sign of primary bone apposition and osteoclast activity around the large nutrient canal would indicate an ongoing reshaping of the nutrient canal overtime, through the development of the animal.

The absence of more complete and articulated remains prevents us to frame this specimen in a developmental series. Nonetheless, the histological features of the specimen here described (presence of primary bone, high superficial cortical vascularization, absence of EFS and a non-completely remodelled cortex) indicate a still growing subadult or an early adult. Lomax et al. [7] mentioned that processes like heterochronic sustained growth rates may have enabled the Late Triassic ichthyosaurs to reach giant sizes (~25+ m). However, more comparative histological data (e.g. juvenile specimens of *Ichthyotitan* and more histological samples of Triassic ichthyosaurs) is needed to identify such developmental strategies in *Ichthyotitan*.

## Conclusions

A large ichthyosaur surangular was collected in 2016 from the UK Upper Triassic and was formally described by Lomax et al. [7] who also reinterpreted the Aust ‘dinosaurian’ bone shafts as belonging to the jaws of giant Triassic ichthyosaurs, something which had also been noted by Huene [27]. Recent support for these assertions was presented by Perillo et al. [17] who further assessed these claims by analysing the bone histology of the Lilstock specimen and the Aust bones. As such, they tested the “*Huene-Lomax hypothesis*” histologically and found that the histology of these bones supports an ichthyosaurian affinity (see also the “*Giant Ichthyosaur hypothesis*” in [18]).

The discovery of another giant ichthyosaur surangular from the Somerset coast of the UK provides additional support for the conclusions presented by [7] and the findings of [17, 18]. The new specimen matches the Lilstock surangular in overall shape, bears the same unique morphology and comes from the same stratigraphic age (Westbury Mudstone Formation, Upper Triassic, latest Rhaetian). The specimen is, however, more complete and better preserved than the Lilstock surangular and includes a large portion of the anterior section, showing distinct facets for the dentary and a section of what is possibly the angular, which appears fused to the surangular. We herein formally assign these two specimens to a new genus and species, *Ichthyotitan severnensis*, which is the first-named giant ichthyosaur from the Rhaetian. This taxon has an estimated body length of around 25 m, or at least somewhere in the 20–26 m range and represents the largest estimate for a prehistoric marine reptile. The histological analysis agrees with similar specimens and advocates for a still growing sub-adult or early adult animal that had yet to reach an asymptotic growth stage. It is, however, worth reiterating that this is based on fragmentary remains and thus more complete specimens are required to confirm the giant size. Furthermore, if the estimates for the Aust bones are correct, as per Lomax et al. [7], then those individuals probably represent the largest ichthyosaurs known.

As summarized by Lomax et al. [7], these giant fragmentary jaw bones may easily be missed or could be mistaken for the remains of dinosaurs because of their size. The authors hoped that the description and identification of the Lilstock specimen would lead to more discoveries, which ultimately led to the identification of the BAS specimen described herein. It is our hope that more complete remains of this enigmatic giant ichthyosaur will be discovered in time. If any additional material is found, we kindly encourage the finders to contact the authors.

## Abbreviations

BRSMG: Bristol Museum and Art Gallery, Bristol, UK
LACM DI: The Dinosaur Institute, Natural History Museum of Los Angeles County, Los Angeles, California, USA
MJML: The Etches Collection Museum of Jurassic Marine Life, Kimmeridge, Dorset, UK
TMP: The Royal Tyrrell Museum of Palaeontology, Alberta, Canada
