# Correction: The last giants: New evidence for giant Late Triassic (Rhaetian) ichthyosaurs from the UK

**DOI:** 10.1371/journal.pone.0317938

**Published:** 2025-01-16

**Authors:** Dean R. Lomax, Paul de la Salle, Marcello Perillo, Justin Reynolds, Ruby Reynolds, James F. Waldron

There is an error in the caption for Fig 4, "Invertebrate and trace fossils found on the bone surface of the BAS surangular, BRSMG Cg3178," panel B. Please see the complete, correct Fig 4 caption here.

**Fig 4:**
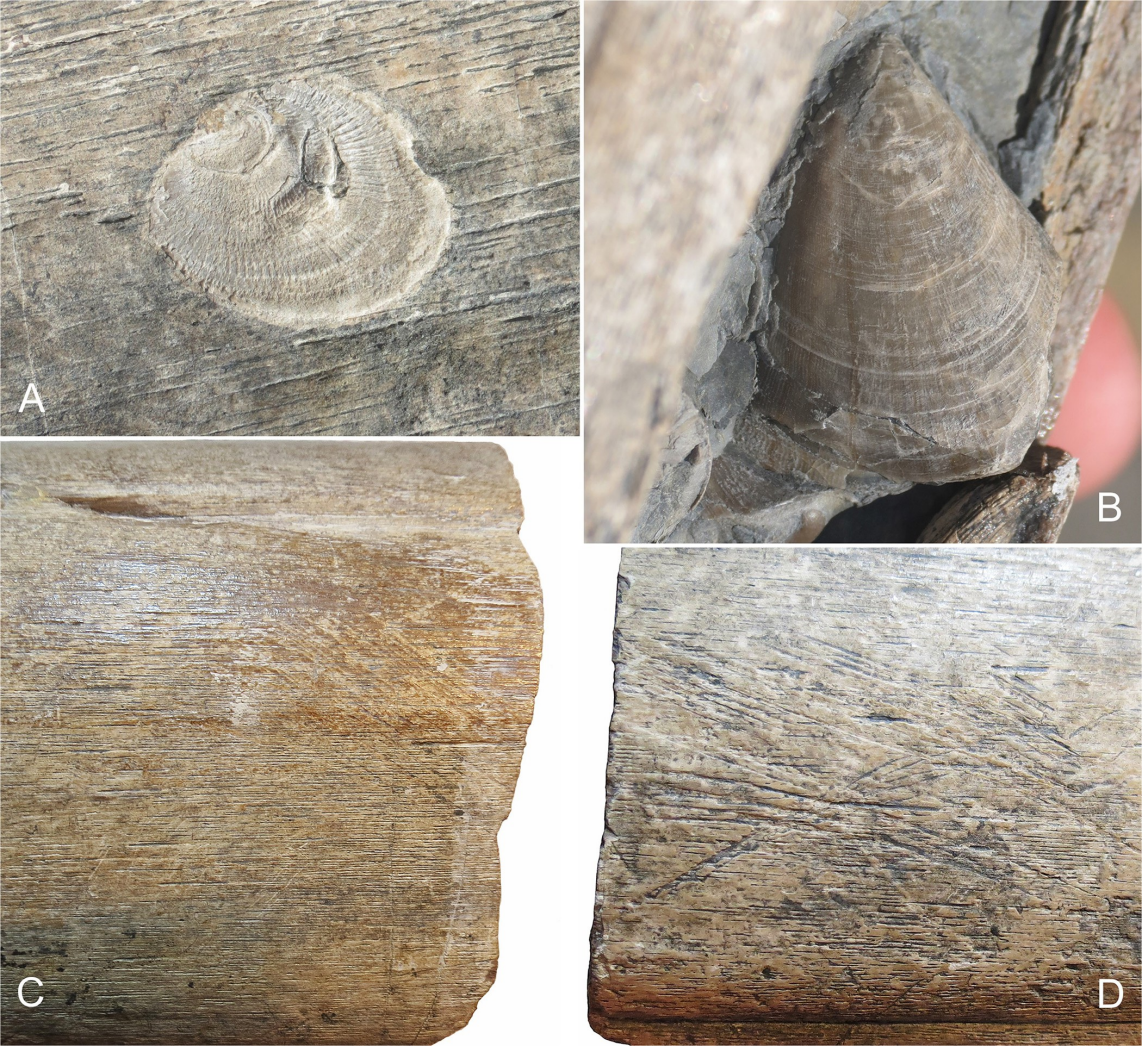
Invertebrate and trace fossils found on the bone surface of the BAS surangular, BRSMG Cg3178. A-B. Associated bivalves, including Atreta intrusstriata (A) and Plagiostoma punctatum (B); it is worth noting that a small group of the latter are preserved adjacent to the coronoid process, see Fig 2C. C-D. Examples of the probable scavenging marks that are also observed in the Lilstock surangular, see Lomax et al. 2018, Fig 4.
